# Ethanol Causes Protein Precipitation—New Safety Issues for Catheter Locking Techniques

**DOI:** 10.1371/journal.pone.0084869

**Published:** 2013-12-31

**Authors:** Gernot Schilcher, Axel Schlagenhauf, Daniel Schneditz, Hubert Scharnagl, Werner Ribitsch, Robert Krause, Alexander R. Rosenkranz, Tatjana Stojakovic, Joerg H. Horina

**Affiliations:** 1 Clinical Division of Nephrology, Department of Internal Medicine, Medical University of Graz, Graz, Austria; 2 Department of General Pediatrics and Adolescent Medicine, Medical University of Graz, Graz, Austria; 3 Institute of Physiology, Medical University of Graz, Graz, Austria; 4 Clinical Institute of Medical and Chemical Laboratory Diagnostics, Medical University of Graz, Graz, Austria; 5 Section of Infectious Diseases and Tropical Medicine, Department of Internal Medicine, Medical University of Graz, Graz, Austria; Institut Pasteur, France

## Abstract

**Objective:**

The ethanol lock technique has shown great potential to eradicate organisms in biofilms and to treat or prevent central venous catheter related infections. Following instillation of ethanol lock solution, however, the inherent density gradient between blood and ethanol causes gravity induced seepage of ethanol out of the catheter and blood influx into the catheter. Plasma proteins so are exposed to highly concentrated ethanol, which is a classic agent for protein precipitation. We aimed to investigate the precipitating effect of ethanol locks on plasma proteins as a possible cause for reported catheter occlusions.

**Methods:**

Plasma samples were exposed in-vitro to ethanol (concentrations ranging from 7 to 70 v/v%) and heparin lock solutions. In catheter studies designed to mimic different in-vivo situations, the catheter tip was placed in a plasma reservoir and the material contained within the catheter was analyzed after ethanol lock instillation. The samples underwent standardized investigation for protein precipitation.

**Results:**

Protein precipitation was observed in plasma samples containing ethanol solutions above a concentration of 28%, as well as in material retrieved from vertically positioned femoral catheters and jugular (subclavian) catheters simulating recumbent or head down tilt body positions. Precipitates could not be re-dissolved by dilution with plasma, urokinase or alteplase. Plasma samples containing heparin lock solutions showed no signs of precipitation.

**Conclusions:**

Our in-vitro results demonstrate that ethanol locks may be associated with plasma protein precipitation in central venous catheters. This phenomenon could be related to occlusion of vascular access devices locked with ethanol, as has been reported. Concerns should be raised regarding possible complications upon injection or spontaneous gravity induced leakage of such irreversibly precipitated protein particles into the systemic circulation. We suggest limiting the maximum advisable concentration of ethanol to 28 v/v% in catheter lock solutions.

## Introduction

Central venous catheters (CVCs) allow life saving interventions by providing reliable venous access for hemodynamic monitoring, fluid and drug administration, hemodialysis, chemotherapy, or total parenteral nutrition in neonate, infant and adult patients. [1]–[3] However, the long-term use of CVCs is fraught with complications including a high rate of infection and thrombus-related dysfunction or occlusion. [4], [5] To mitigate the impact of these complications, anticoagulative, antibiotic or antimicrobial catheter lock solutions have been used prophylactically or therapeutically to maintain the intraluminal patency of CVCs. [6] Ethanol, considered an alternative to heparin lock solution for CVCs, is an antimicrobial agent currently used predominantly in patients with intestinal failure relying on total parenteral nutrition as well as in patients with chronic kidney failure requiring renal replacement therapy. [7]–[8] In-vitro and in-vivo studies have shown the efficacy of ethanol at varying concentrations (20–74%) to eradicate various planktonic pathogens as well as microbial organisms embedded in the intraluminal biofilm of indwelling CVCs. [9]–[13] In 2011, the latest guidelines from the Centers for Disease Control and Prevention discussed, but did not recommend ethanol locks (ELs) to prevent intravascular catheter related bloodstream infections (CRBSI), while the American Pediatric Surgical Association concluded that ELs can be administered safely and can effectively reduce the incidence of CRBSI (Grades A/B recommendations). [14]–[15] Although Oliveira et al. [7] and Wolf et al. [16] stated in recently published reviews that the evidence for the effectiveness of EL in CRBSI prevention is robust, they were nonetheless concerned about adverse thrombotic events, retrospective evidence and the absence of a systematic safety assessment in the studies covered.

Instillation of the listed filling volume of lock solutions, such as ELs, into the lumen of a CVC was believed to ‘lock’ the CVC, suggesting that the full injected volume of the locking anticoagulant remained inside the CVC. Nonetheless, leakage of catheter lock solutions into the systemic circulation of approximately 20–25% at the time of instillation has been demonstrated repeatedly. [17]–[18] Several authors reported increased aPTT times or bleeding episodes if heparin was instilled to lock CVCs. [19]–[21] The spillage during instillation of the listed filling volume into the CVC is a physical consequence of parabolic rather than plug flow distribution within the catheter and thus cannot be avoided. Furthermore, gravity induced loss of lock solution into the systemic circulation should be considered when lock solutions (e.g. ethanol) with densities different from whole blood are used. [22]–[23] In this case, depending on the density gradient between the lock solution and blood, this seepage of lock solution is accompanied by a concomitant blood inflow into the CVC until equilibrium is achieved. [24] Several studies using ELs for prevention or treatment of CRBSI reported either CVC occlusion or precipitated material as well as clots clearly visible upon line aspiration although the underlying mechanisms remained unclear. [25]–[28] We recently published similar observations in CVCs used with hypertonic citrate lock solution, a protein precipitating agent. [29], [30] For decades, plasma proteins have commonly been purified by precipitating them with organic solvents, mainly ethanol. [31] Precipitated protein might cause both CVC occlusion and micro-embolism through small protein emboli released from the CVC spontaneously or, particularly, when the lock is flushed into pulmonary circulation. [29], [32].

The objective of this in-vitro study was to investigate the conditions for protein precipitation in ethanol locked CVCs. The known interaction of ethanol with human erythrocytes mediates hemolysis. [33] Since hemolysis precludes visual detection of precipitates in test tubes with any accuracy, plasma had to be substituted for whole blood, and so human plasma was exposed to ethanol in varying concentrations to assess protein precipitation. In an experimental setting designed to mimic different in-vivo situations, the material in the catheter was analyzed after EL lock instillation.

## Methods

### Ethics Statement

The study was approved by the Ethics Committee of the Medical University of Graz, Austria (registration number: 25–260 ex 12/13). Written informed consent was obtained from the blood donors for the dilution studies.

### Dilution Studies

The effect of ethanol on protein precipitation was studied by mixing four parts of aqueous ethanol solution prepared from medical grade ethanol (Department of Hospital Pharmacy, Medical University of Graz, Austria) in concentrations ranging from 7 to 70 vol% with one part of plasma or serum. Mimicking the in-vivo process of plasma entry into an ethanol filled CVC, one ml of plasma or serum was transferred into plain sample tubes containing four ml of ethanol in varying concentrations. All the samples then underwent standardized investigation for precipitation after 20 min, 24 h and 48 h of incubation as described below. Tests were repeated in triplicate at room temperature (24°C) and at body temperature (37°C). The same experimental design was used to study the effect of heparin lock 1000 I.U./mL, 5000 I.U./mL and 10000 I.U./mL on plasma proteins. Fresh human plasma or serum from two healthy adult male donors was obtained by centrifugation of lithium-heparin, EDTA or citrate (Vacuette®, Greiner Bio-One, Austria) anticoagulated (or coagulated) whole blood at 4000 rpm for 10 min (Eppendorf centrifuge 5810R, Germany) for the test series.

### Standardized Investigation for Precipitation

The plasma or serum ethanol as well as the plasma (or serum) heparin mixtures were centrifuged at room temperature (24°C) or at body temperature (37°C) and 4000 rpm for 10 min (Eppendorf centrifuge 5810R, Germany). Precipitation was assessed with a visual score, ranging from +++ (much) to – (none). Reversibility of protein precipitation was determined by visual inspection after addition of 20 ml each of plasma, urokinase 50000 Units (Urokinase®, Medac Gmbh, Germany) or alteplase 5 mg (Actilyse®, Boehringer Ingelheim Pharma, Germany) to dried precipitate and gentle mixing for 10 min.

### Protein Analysis of Precipitates

Precipitated proteins were analyzed using sodium dodecylsulfate polyacrylamide gel electrophoresis (SDS-PAGE) followed by silver staining. Proteins were resolubilized in 25 mM ammonium bicarbonate containing 8 M urea and 2.5% SDS (pH 7.4). Whole protein content was determined with a bicinchoninic acid protein assay (Pierce, Thermo Fisher Scientific, Rockford, IL, USA). After addition of 0.05 mL sample buffer (NuPAGE LDS, Invitrogen, Carlsbad, CA, USA) samples were heated at 95°C for 5 minutes. Six micrograms of proteins were electrophoresed on 4% to 12% gradient sodium dodecyl sulfate–polyacrylamide gel electrophoresis (SDS-PAGE) gels (Novex pre-cast gels, Invitrogen, Carlsbad, USA) along with an appropriately sized standard (SeeBlue Plus 2, Invitrogen, Carlsbad, USA) in 2-(Nmorpholino) ethanesulfonic acid SDS running buffer. Silver staining was performed with a commercially available kit (Pierce, Thermo Fisher Scientific, Rockford, IL USA).

### Catheter Studies

**Figure 1:**
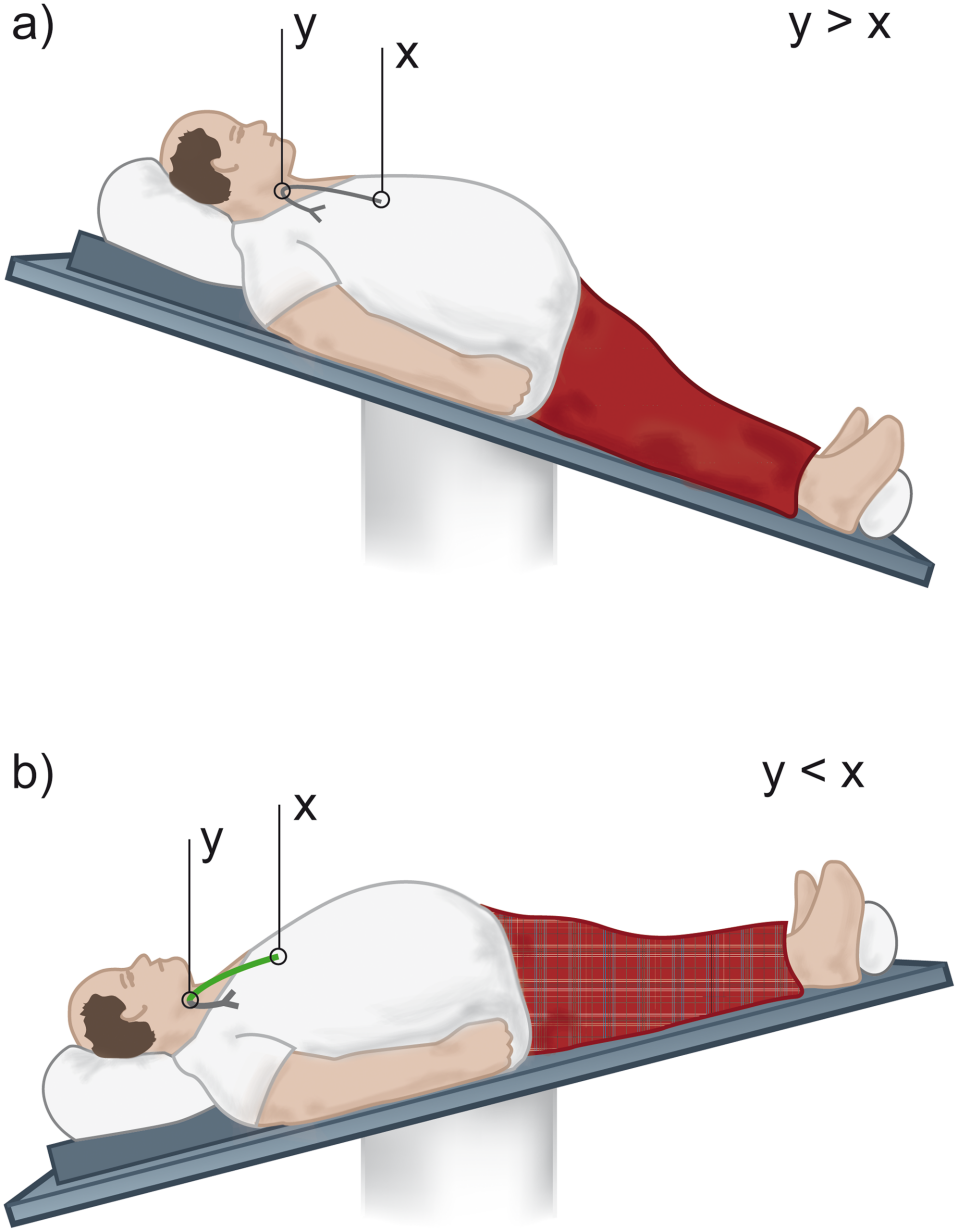
Relative catheter positions and plasma influx. a) If the venous insertion point (y) is higher relative to the tip (x), there is no plasma or in-vivo whole blood influx b) If the venous insertion point is lower (y) relative to the tip (x) plasma or in-vivo whole blood enters the lumens of the catheter (as indicated by green colour). The same relative catheter position can be assumed for patients in upright position with femoral catheters.

In-vitro, catheter precipitations were studied using ethanol lock solutions in concentrations ranging from 7 to 70 vol%. Hemodialysis catheters (Medcomp® Split Cath® III 14FR/32 cm, Medical Components Inc., Harleysville, USA; GamCath™ HighFlow Dolphin® Protect Double Lumen catheter 250 mm, Gambro, Hechingen, Germany) were flushed and filled with saline 0.9%. The tip of the primed catheter was placed into a bag filled with citrated fresh frozen plasma (Department of Blood Group Serology and Transfusion Medicine, Medical University of Graz, Austria) and kept at 37°C in a thermostat bath. Beforehand, the plasma was colorized with a fluorescent green dye (indocyanine green, ICG-Pulsion, Pulsion Medical System AG, Munich, Germany) that binds to plasma proteins. The catheter was then locked using the exact volume of lock solution indicated on each catheter port. Catheters were positioned in three experimental setups to mimic different in-vivo situations (Figure 1). First, the catheter was locked and left in upright position for the incubation period, simulating a jugular (or subclavian) placed catheter in permanent vertical position. Second, following lock instillation in upright position the catheter was tip elevated relative to its insertion point into the jugular (or subclavian) vein to simulate a recumbent or head down tilt situation. Third, to simulate femoral placement, the lock was applied in upside-down position and the catheter remained in that position for the incubation period. After 20 min of incubation all material in the catheter (‘lock’) was recovered by emptying its contents into a plain sample tube. The samples were visually inspected for dye that would indicate influx of plasma into the catheter due to gravity (density gradient) induced spillage of lock solution. Standardized investigation for precipitation was performed as described above. Measurements were repeated in triplicate at room temperature (24°C) for each catheter and for each lock solution. A sample of colorized and non-colorized plasma was visually assessed and investigated for dye induced protein or mineral precipitation prior to contact with ethanol. Also, to test for possible interactions between ethanol and citrate, mixtures in varying concentrations underwent standardized investigation for precipitation.

## Results

### Dilution Studies

**Figure 2:**
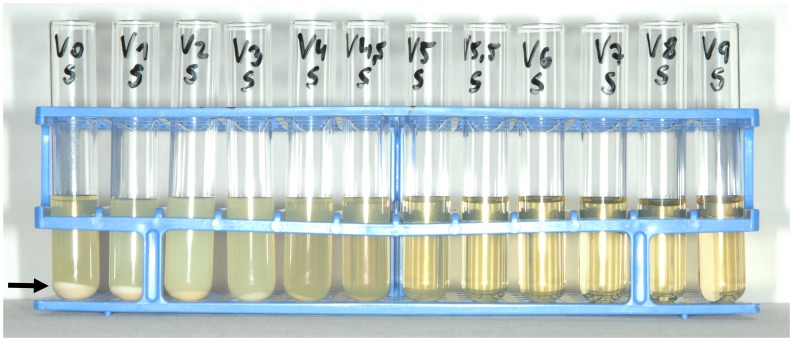
Dilution studies. The arrow indicates the precipitated protein within the tube (V0) after centrifugation of the test solution consisting of 1 mL plasma and 4 mL ethanol 70%. Concentrations decrease from left to right. Tubes with test solutions containing ethanol locks of ≤28% in the dilution series (V6 toV9) revealed no signs of precipitation. The test series was performed at room temperature (24°C).

Using different concentrations of EL solutions (7 to 70 vol%), protein precipitation was detected in samples containing ELs above a concentration of 28% in all test series performed at room and at body temperature (Table 1, Figure 2). The precipitate could not be re-dissolved by the addition of plasma, urokinase or alteplase independent of incubation time. Samples containing heparin lock solution showed no signs of precipitation.

**Table 1:** Results of in-vitro protein precipitation tests (dilution studies).

Test solution^a^		Ethanol concentration of lock solution [%]^b^	Visible precipitation^c^
V0	Ethanol/Plasma^d^	70	+++
V1	Ethanol/Plasma^d^	63	+++
V2	Ethanol/Plasma^d^	56	++
V3	Ethanol/Plasma^d^	49	++
V4	Ethanol/Plasma^d^	42	+
V4.5	Ethanol/Plasma^d^	38.5	+
V5	Ethanol/Plasma^d^	35	+
V5.5	Ethanol/Plasma^d^	31.5	+
V6	Ethanol/Plasma^d^	28	–
V7	Ethanol/Plasma^d^	21	–
V8	Ethanol/Plasma^d^	14	–
V9	Ethanol/Plasma^d^	7	–

^a^ Test solutions consisted of 1 ml plasma (or serum) and 4 ml ethanol lock solution (V0– V9, concentrations ranged from 70 to 7%).

^b^ mimicking the conditions inside the catheter. Tests were conducted at room and body temperature.

^c^ Precipitation was assessed with a visual score, ranging from +++ (much) to – (none).

^d^ Blood sample characteristics: hematocrit 0,42; albumin 4.5 g/dl; total protein 6.5 g/dl.

### Protein Analysis of Precipitates

**Figure 3:**
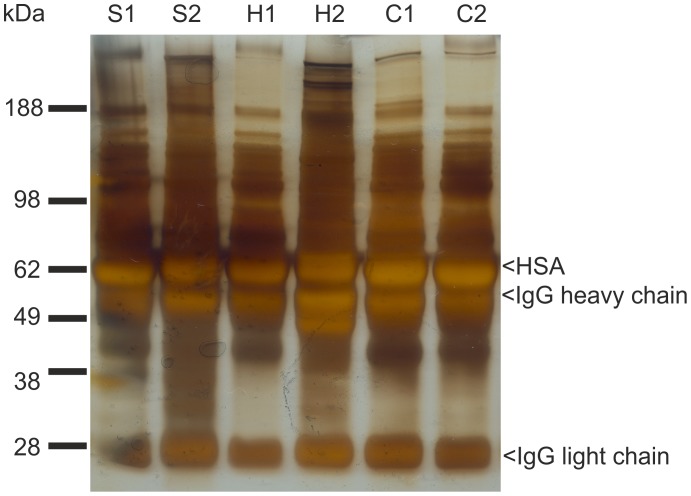
Protein analysis of precipitates. Representative silver-stained SDS-PAGE gel of protein precipitates. Lanes correspond to serum precipitated with 70% ethanol (S1) or 35% ethanol (S2), lithium-heparin plasma precipitated with 70% ethanol (H1) or 35% ethanol (H2), and citrate plasma precipitated with 70% ethanol (C1) or 35% ethanol (C2).

SDS-PAGE revealed a complex mixture of proteins in all samples with human serum albumin and IgG constituting the most prominent bands (Figure 3). High-abundance protein fractions did not vary significantly between precipitates from serum, or from samples containing lithium-heparin and citrate. Proteins smaller than 25 kDa were not present. No differences could be observed in the protein profiles precipitated with 35% or 70% ethanol.

### Catheter Studies

**Figure 4:**
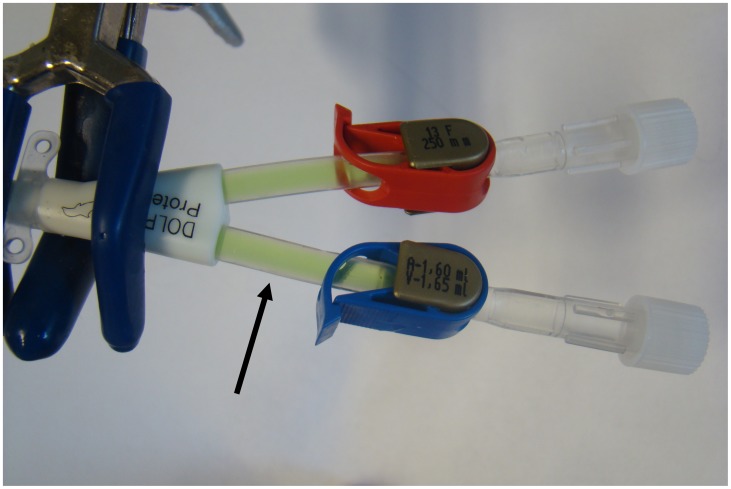
Catheter studies. After ethanol instillation (concentrations ranging from 7 to 70%) in upright position, a simulated jugular (or subclavian) catheter contained green colored plasma up to the clamps (see arrow) after the catheter tip was elevated into recumbent/head down tilt position. Gravity forced ethanol lock to leak out of the catheter followed by plasma, or in vivo whole blood influx, respectively.

All catheter contents (‘locks’) recovered from experiments where the tip of the catheter was elevated relative to its assumed insertion point into the vein, initially (femoral placed catheter) or after change into recumbent/head down tilt position (jugular or subclavian placed catheter), contained green dye indicating a gravity (density gradient) driven plasma influx into the catheter (Table 2, Figure 4). Throughout all test series protein precipitation was detected in dyed catheter contents from catheters filled with lock solutions containing ethanol above a concentration of 28 vol% (Table 2). The precipitates could not be re-dissolved by the addition of plasma. In contrast, catheter contents from simulated jugular (or subclavian) catheters in permanent vertical position showed neither color changes indicating plasma influx nor signs of precipitation (Table 2). Samples of colorized and non-colorized plasma prior to contact with ethanol as well as ethanol/citrate mixtures showed no signs of precipitation.

**Table 2:** Results of catheter content (‘lock’) analysis in varying catheter positions.

Ethanol concentration of lock solution [%]	Visible precipitation^a^ jugular-vertical^b^	Visible precipitation^a^ jugular-recumbent^c^	Visible precipitation^a^ femoral-vertical^c^
70	–	+++	+++
63	–	++	++
56	–	++	++
49	–	+	+
42	–	+	+
38.5	–	+	+
35	–	+	+
31.5	–	+	+
28	–	–	–
21	–	–	–
14	–	–	–
7	–	–	–

^a^ The centrifuged catheter content was assessed for precipitation with a visual score, ranging from +++ (much) to – (none).

^b^ No green staining or precipitation was observed in catheter content samples in jugular (subclavian)-vertical position.

^c^ All catheter content (‘lock’) samples of jugular (subclavian) recumbent/head down tilt or femoral vertical position showed green colour indicating plasma influx into the catheter. Precipitation was only present if ethanol lock solutions were used with concentrations above 28%. Plasma sample characteristics: total protein 5.3 g/dL, albumin 3.1 g/dL and density 1.0197 g/cm^3^ at 37°Celsius.

## Discussion

Guidelines for the clinical use of antimicrobial lock solutions call for tests to exclude visual precipitation that occurs when certain antibiotics are mixed with anticoagulants. [34] Precipitation observed in solutions to be administered intravenously or in locking anticoagulants for CVCs is considered to be both pharmacologically and physically incompatible. [35], [36] In clinical practice, lock solution is always partly spilled into the systemic circulation when instilled and often has to be injected completely if aspiration is not feasible. [17] The current standard to obviate precipitation reactions in agents used as anticoagulants for CVCs focuses solely on the pure lock solution. [37], [38] However, this approach might be inadequate if the fluid density of the analyzed lock solution is different from blood. Evaluation of such lock solutions, as recently shown for trisodium citrate, should include in-vitro tests in the presence of blood or plasma to rule out protein precipitation. [24], [29] We performed in vitro analyses to demonstrate that intraluminal protein precipitation might occur if ethanol is instilled in CVCs at concentrations above 28% (Table 1,2; Figure 1), whereas heparin has been proven not to cause precipitation. Our results are supported by previous studies about plasma protein precipitation by ethanol. [39], [40] Analysis of the sediments by SDS-PAGE revealed a mixture of several proteins with the most abundant plasma proteins, with human serum albumin and IgG as the dominant fractions in all samples (Figure 3). This finding argues for a non-specific precipitation of plasma proteins. The lack of proteins smaller than 25 kDa in the sediments suggests that these proteins are less prone to ethanol precipitation. Interestingly, differing ethanol concentrations only affected the overall amount of proteins precipitating from plasma, while types and relative amounts of proteins remained comparable. Additionally, no significant differences in protein amounts or patterns were seen with serum, lithium-heparin, and citrate, speaking against a potential influence of anticoagulants on the precipitation process. Recently, it has been shown that a lock solution containing 7% citrate, 20% ethanol and 0.01% glyceryl trinitrate is able to fully eradicate biofilm organisms in vitro. [41] Therefore, ethanol is potentially effective in reducing CRBSI incidence in concentrations where plasma protein precipitation is absent, although this has not yet been targeted in clinical trials. In general, spillage of lock solution has two underlying mechanisms: (1) unavoidable spillage due to parabolic flow distribution inside the CVC during injection (20–25%) even of the listed filling volume and (2) gravity induced seepage out of the CVC due to differences in fluid density between blood and lock solution. [17], [22], [23], [29] With regard to point one, the amount of ethanol administered systemically during injection is currently neglected. Since only mild and transient symptoms like fatigue, headache, dizziness, alcohol taste, drowsiness or nausea are generally attributed to ethanol injection, ELs have been regarded safe and non-toxic. [12], [42], [43] With regard to point two, the fluid densities of blood (∼1.05 g/cm^3^) and plasma (∼1.02 g/cm^3^) are higher compared to ethanol (0.853 g/cm^3^ ethanol 70%; 0,943 g/cm^3^ ethanol 30%) at 37°Celsius. [44], [45] While the spillage during injection (20–25%) is unaffected by the catheter position, the density (gravity force) induced spillage is greatly affected by the relative catheter position. In any patient position with the tip of the CVC elevated relative to the insertion point into the vein, which might occur even during sleep, gravity forces EL to leak out of the CVC to be replaced by blood (Figure 1, Figure 4). Consequently, when plasma proteins are exposed to ethanol, intraluminal precipitation occurs instantly at concentrations above 28%, as shown by our dilution studies and catheter experiments which were performed at room and body temperature to cover the possible temperature range alongside the catheter. (Figure 2, Figure 4). At temperatures above 10°Celsius the precipitate cannot be re-dissolved due to protein denaturation and might cause catheter occlusion. [46] The mentioned fluid exchange is governed by laws of hydraulics that allow conclusions for clinical practice to be drawn. [47].

In patients with CVCs in jugular or subclavian position, the phenomenon described above can only be seen under a certain condition. Between EL instillation and removal, the patient just once has to be in recumbent or head down tilt position with the tip of the catheter elevated relative to its venous insertion point, as in Trendelenburg-position due to hypotension after hemodialysis or if the patient is lying flat (Figure 1). The amount of blood influx and subsequent precipitation differs in response to the time spent in these positions. In contrast, in patients with femoral catheters blood will always enter the lumens of the CVC until equilibrium is reached. Accordingly, in femoral CVCs a higher occlusion rate might be expected.

Ethanol (96%) is approved for sclerotherapy of peripheral venous malformations. It is pharmacologically active, inducing instant thrombosis and destruction of the vascular wall to the level of the intima by denaturating plasma and endothelial cell proteins. [48], [49] Plasma proteins are brought into contact with ethanol, forming a precipitate whenever the lock is aspirated, regardless of CVC insertion site. This is due to the parabolic flow profile of blood towards the Luer end and incomplete ethanol removal from the catheter wall. *Wales et al.* [27] stated ‘if the ethanol is withdrawn, there is potential risk for clotting of the CVC and sclerosis of the vein at the catheter tip’. In pediatric patients on total parenteral nutrition a standardized procedure to minimize systemic effects revealed difficulties in withdrawing ELs (70–100%), as well as vascular access occlusion or visible line thrombosis. [25], [26], [28], [50] The vast majority of studies suggest flushing ELs at the end of dwell time to prevent clotting inside the catheter, most likely because ethanol is considered to resolve lipid residues in CVCs used for total parenteral nutrition, although the underlying rationale was not given. [12], [27], [39], [43], [51]–[55] Introduction of irreversibly precipitated protein particles into the systemic circulation might occur not only when the ethanol lock solution is injected as a bolus, but also following every single instillation of ethanol due to gravity induced leakage of precipitate, thus indicating physical incompatibility of high ethanol concentrations. Several trials, though mainly retrospective, reported no unforeseeable adverse events or higher occlusion rates of CVCs that could be attributed to the ethanol locking solution. [12], [43], [51], [56], [57] Other studies, however, showed CVC occlusions, which in one case was associated with device fracture, as well as adverse events after EL instillation or flushing through. [25], [27], [42], [50], [52] One prospective study was even suspended prematurely due to a high CVC occlusion rate. [28] These studies, however, did not include CVC occlusion rates or adverse events as primary or secondary outcome measures and therefore the absolute risk of occlusion associated with ethanol lock use is unknown, but may be related to protein precipitation. A risk-benefit analysis should be performed between the risk of introducing precipitate into the blood stream or higher catheter occlusion rates compared to potential benefits such as CRBSI prevention to determine the future direction in using higher concentrations of ethanol for catheter locking.

There are important limitations to our in-vitro approach that deserve mention. Firstly, due to the hemolytic effects of ethanol and knowing that ethanol targets plasma proteins, we used plasma to assess precipitation. Since the density of plasma is slightly lower than that of blood, the in-vitro situation might have resulted in less plasma influx into the CVC and less precipitation than in vivo. Secondly, plasma for dilution and catheter studies was obtained from healthy adult donors to counteract interindividual differences in healthy volunteers. However, possible alterations in the plasma status of critically ill patients might affect protein precipitation. Thirdly, precipitation was assessed visually and very small amounts might have been missed.

### Conclusion

Our in-vitro results demonstrate that high ethanol concentrations used in catheter locks might induce plasma protein precipitation in CVCs. To avoid possible occlusion of vascular access devices and introduction of irreversibly precipitated protein particles into the systemic circulation, we suggest limiting the maximum advisable concentration of ethanol to 28 v/v% in catheter lock solutions. Furthermore, any lock solution containing plasma precipitating agents with density characteristics different from blood, either more or less dense, should be evaluated for protein precipitation prior to clinical use.
